# Post-COVID isolated subclavian artery dissection with multiple cerebral infarctions

**DOI:** 10.1186/s41983-022-00549-8

**Published:** 2022-10-01

**Authors:** Taha K. Alloush, Adel T. Alloush, Fayez Marzouk, Khaled O. Abdulghani, Hossam M. Shokri

**Affiliations:** 1 Department of Neurology and Psychiatry, Faculty of Medicine, Ain Shams University, Cairo, Egypt; 2 Department of Geriatrics and Gerontology, Faculty of Medicine, Ain Shams University, Cairo, Egypt; 3 Departement of Vascular Surgery, The Good Shepherd, Italian and alHaya Hospitals, Cairo, Egypt; 4 Department of Neurology and Psychiatry, Helwan University School of Medicine, Cairo, Egypt

**Keywords:** Subclavian dissection, COVID-19, Limb ischemia, Cerebral infarction

## Abstract

**Background:**

Coronavirus disease 2019 (COVID-19) is a pandemic disease which predominantly affects the respiratory system with high critical care mortality and morbidity, yet it also causes multiple organs dysfunction in affected patients. There is a strong evidence that it increases the susceptibility of cerebrovascular strokes in such patients. Besides this prothrombotic complication, arterial dissection can be one of its mechanisms increasing the risks of stroke.

**Case presentation:**

Herein, we report a case of spontaneous isolated subclavian artery dissection in a COVID-19 patient. Sixty-one-year-old female presented with spontaneous isolated subclavian artery dissection without any traumatic events nor history of connective tissue disorders. She had left upper limb ischemia followed by cerebellar, thalamic and occipital infarctions. Whether this patient’s subclavian artery dissection was triggered by exaggerated inflammatory response or arteriopathy secondary to COVID-19 remains speculative.

**Conclusions:**

Nonetheless, arterial dissection can be one of its complications, it is essential for treating physicians to be attentive for the diversity of COVID-19 clinical manifestations.

## Background

Isolated subclavian artery dissection is a rare condition, and generally related to arterial catheterization, connective tissue disease or blunt trauma. Yet, spontaneous dissection without any traumatic events has been rarely reported in the medical literature [1]. Moreover, there are very few case reports with spontaneous subclavian artery dissection causing neurological deficits due to ischemic stroke. The main clinical manifestations of subclavian artery dissection are chest, back, and neck pains, arm pulselessness, dizziness with nausea and vomiting and visual disturbances [2–7]. The complications of subclavian artery dissection may include ischemic stroke, arm ischemia and subclavian steal syndrome [2, 7, 8]. As typical presentation is sudden onset of severe, ripping chest pain that can be very similar to aortic dissection; both of which can be life-threatening conditions but in different degrees [9].

Coronavirus disease 2019 (COVID-19) is a pandemic disease with eminent worldwide health risk. COVID-19 initially affected older population with co-morbidities; however, it is widespread manifestations in all age-groups are being reported widely [10, 11]. Predominantly, the respiratory system is usually affected; however, other systems are not spared [10].

There is a clear evidence that patients without significant risk factors are presenting with strokes, since this pandemic has started [12]. Although, the true relationship between COVID-19 and stroke incidence remains under investigation, there have been many reports in the literature concluding that COVID-19 can produce a prothrombotic state that results in thromboembolic complications [13]. Coagulopathy and vascular endothelial dysfunction resulting from the “cytokine storm”; an exaggerated systemic inflammatory state, have also been suggested [14, 15]. Moreover, direct SARS-CoV-2 viral invasion of the vascular endothelial cells using their surface angiotensin-converting enzyme 2 (ACE2) receptors can also result in arterial dissection [16, 17].

There are many case reports describing the patients who presented with spontaneous arterial vascular dissection [18–22]. Whether the increased tendency and the pathological mechanism behind dissection is triggered by COVID-19 is still vague, it does require thorough observation and investigations. Hereby, we report the first case, to our knowledge, of spontaneous isolated subclavian artery dissection in a post-COVID-19 patient.

## Case presentation

A 61-year-old woman with diabetes and history of COVID-19 infection 3 weeks prior to presentation, was admitted to the emergency department on August 10, 2021, with a sudden onset of with severe pain left upper limb absent pulses, pallor, color changes of left hand, fixed mottling index and middle fingers. There was no history of trauma or neck manipulation. Initial electrocardiogram and Transthoracic echography were normal. The patient’s initial blood investigations were not significantly abnormal, with a negative PCR result for COVID-19.

The patient immediately underwent Duplex imaging, that showed acutely thrombosed and distended left ulnar artery just after its origin by few centimeters in the forearm with damped monophasic flow in the proximal segment and no flow in the rest of its segment till wrist level. Acutely thrombosed and distended left radial artery at the wrist level and its segmental course in the snuff box and hand (flow PSV nearly 5 cm/s) was also noted. The radial artery in its course in forearm showed poor filling with weak monophasic flow. Left brachial artery was patent with low PSV pattern.

Attempted thrombectomy from brachial artery was done first under local anesthesia then under general anesthesia due to severe pain. This was followed by exposure of radial and ulnar arteries just above her left wrist. Arteries filled with old, organized thrombi with no back flow. Next day nerve block was done with chemical symathectomy to improve to improve flow. Follow-up Duplex showed the subclavian, axillary and brachial arteries appear were seen patent with biphasic pattern and with no areas of segmental occlusion. The upper two-thirds of the left ulnar artery was seen patent with irregular lumen; however, its distal segment is seen attenuated and occluded. The lower two-thirds of the left radial artery was also seen attenuated and occluded.

**Fig. 1:**
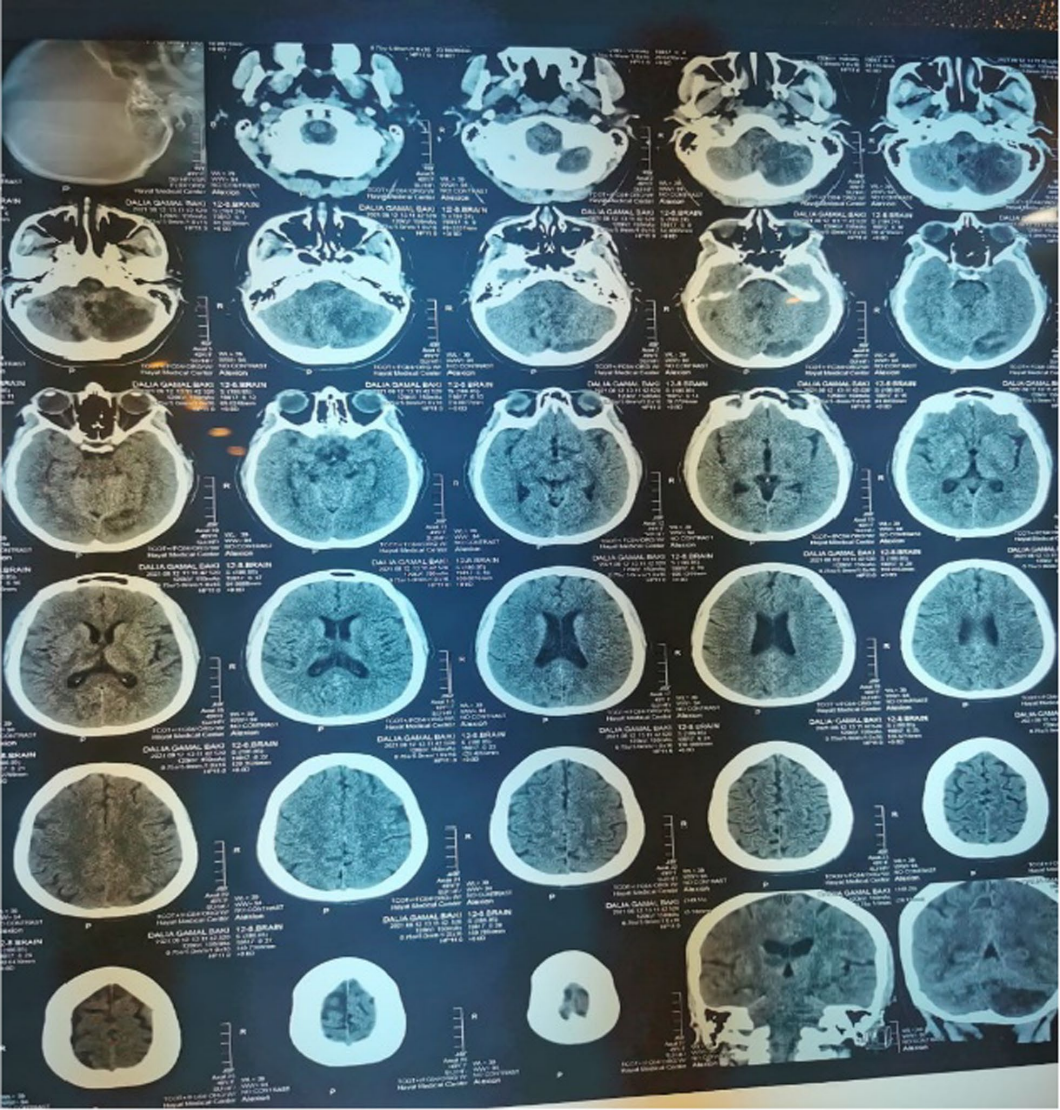
CT of the brain was done and showed left cerebellar, left occipital as well as right thalamic recent infarctions

**Fig. 2:**
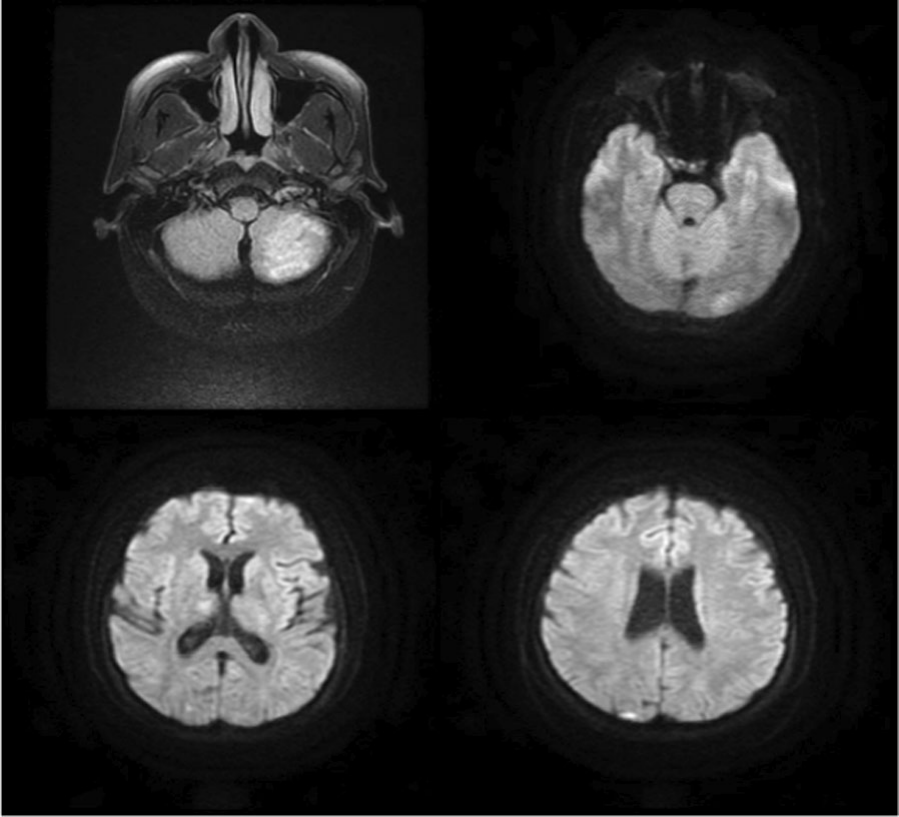
MRI brain axial DWIs show left inferior cerebellar, left occipital, and right thalamic subacute infarcts and right occipital acute cortical infarct

On the third day, the patient had severe cervical and occipital pain with radiation to the left arm, repeated attacks of vertigo and vomiting, heaviness of the left upper limb with limb dysmetria and visual field defect. Computed tomography (CT) and MRI of the brain were done and showed left cerebellar, left occipital, right thalamic and right occipital recent infarctions (Figs. 1 and 2).

**Fig. 3:**
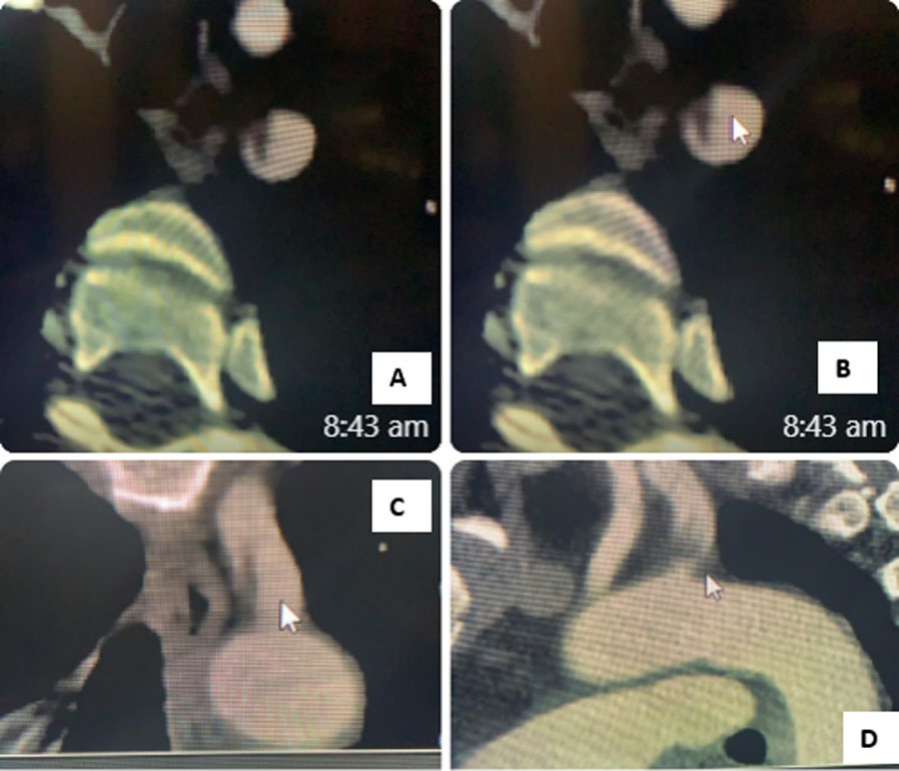
Multi-slice CT angiography **A** and **B** axial, **C** coronal and **D** sagittal images that demonstrate a dissection flab at the origin of left subclavian artery

128^multislice^ CT Angiography of aortic arch, neck arteries, carotid and cerebral arteries was done and showed aortic arch appears to the left side with normal arrangement of its branches. The left subclavian artery showed focal dissection at its origin with intimal flap extending for a distance about 17 mm, and the vertebral arteries appeared well opacified bilaterally up to the skull base and the basilar artery. Otherwise, no significant CT angiography of the intra and extracranial arterial circulation (Fig. 3). The patient was put on full dose anticoagulation.

During the patient’s whole emergency stay, he rather remained hemodynamically and clinically stable, with no new symptoms reported apart from those reported during the third day of admission, and she is currently being followed up with the neurologist, geriatrician, and vascular surgeon in the outpatient department.

## Conclusions

In this report, we describe a 61-year-old female patient with spontaneous subclavian artery dissection. The patient reported no significant traumatic events nor history of connective tissue disorders, systemic arteriopathy, or other contributing factors. Whether this subclavian artery dissection was actuated by the inflammatory response or arteriopathy secondary to SARS-CoV-2 infection remains unproven.

Cervicocranial arterial dissection is one of the known etiologies for ischemic stroke among adults. In a study conducted be Oxley and colleagues, they reported a case series of COVID-19 patients, without significant medical history presenting with stroke due to a large vessel occlusion [12]. With more COVID-19 patients presenting with strokes, arterial dissection could be one of the pathological mechanisms. Arterial dissection in COVID-19 patients could be due to exaggerated inflammatory response causing serious endothelial dysfunction [14]. Another possible pathological mechanism is the direct SARS-CoV-2 viral invasion of the vascular endothelial cells. SARS-CoV-2 uses its surface spike proteins to bind to the ACE2 receptors, the widely expressed receptors in the vascular endothelium throughout the body which in turn can increase probability of vascular endothelial injury in COVID-19 patients [17].

Possible causes of cerebral stroke in such case, in the absence of vertebral artery dissection, may include subclavian intramural hemorrhage, false aneurysm, thrombosis, or emboli to the head and neck or left upper extremity [23]. In this case the multiple cerebellar, thalamic and occipital infarctions of different ages favor embolism as a possible mechanism.

Arterial dissection can be an unusual SARS-CoV-2 complication that treating physicians need to be aware of. The management plan for such patients must be tailored in light with the clinical evaluation and imaging studies. Therefore, it is recommended that vascular imaging of the head and neck must be added to the work-up in COVID-19 patients with cerebrovascular stroke. Similarly, these vascular imaging may also be considered in COVID-19 patients who present with unexplained and resistant headaches especially if accompanied with neck pain. Moreover, patients presenting with arterial dissection even in the absence of cerebrovascular stroke must be screened for SARS-CoV-2 infection. Although a distinctly rare entity, subclavian artery dissection should be remembered in the differential diagnoses of cerebrovascular stroke.

COVID-19 pandemic is still going on with frequent mutations and variants. It is for sure a deceiving disease that totes many medical challenges, including hypercoagulopathy, endotheliopathy and arterial dissection especially in its severe cases. Critical thrombotic events especially in critically ill patients, have been described extensively. Physicians must pay meticulous attention in that regard to diagnose them as early as possible. Finally, we recommend that vascular imaging of the head and neck must be added to the work-up in COVID-19 patients with cerebrovascular stroke.

## Data Availability

The data sets generated and analyzed during the current study are not publicly available due to institutional limitations, yet they are available from the corresponding author on reasonable request.

## Abbreviations

ACE-2: Angiotensin-converting enzyme 2
COVID-19: Coronavirus disease 2019
SARS-CoV-2: Severe acute respiratory syndrome coronavirus-2
